# Echocardiographic Findings in Infants of Diabetic Mothers

**DOI:** 10.7759/cureus.92493

**Published:** 2025-09-16

**Authors:** Ahmed Samir, Carmen Nassar, Ghada M Idriss, Hanaa Abou-elyazid, Saif A Alzahrani

**Affiliations:** 1 Department of Pediatrics and Neonatology, King Fahad General Hospital, Al-Baha City, SAU; 2 Department of Community Medicine, Faculty of Medicine, Al-Azhar University, Cairo, EGY; 3 Department of Pediatrics and Neonatology, King Fahad Hospital, Al-Baha City, SAU

**Keywords:** congenital heart defects, echocardiographic findings, gestational diabetic mothers, infants of diabetic mothers, neonate

## Abstract

Background

Maternal diabetes mellitus (DM) is a well-established risk factor for congenital heart disease (CHD) in neonates. Despite advances in perinatal care, infants of diabetic mothers (IDMs) remain at increased risk for both structural lesions and functional cardiac changes.

Objective

To determine the prevalence and spectrum of echocardiographic abnormalities in neonates of diabetic mothers and compare them with matched controls.

Methods

A retrospective analytical study was conducted at King Fahad Hospital, Al Baha, from January 2024 to June 2025. Two hundred neonates (100 IDMs, 100 controls) underwent standardized echocardiographic assessment within the first week of life. Maternal and neonatal data were reviewed, and statistical analyses were performed to assess associations with clinical variables.

Results

Pathological CHDs were detected in 24% of IDMs, most commonly ventricular septal defect, patent ductus arteriosus, and hypertrophic cardiomyopathy. Transitional findings such as patent foramen ovale and small patent ductus arteriosus (PDA), considered physiological at this age, were present in 64%, while only 12% had completely normal echocardiograms. Lower birth weight-for-gestational-age was significantly associated with CHD (*p* = 0.026). No consistent differences were observed by maternal diabetes type or management modality.

Conclusion

IDMs are at significantly higher risk of structural congenital cardiac anomalies compared with controls, irrespective of maternal diabetes type. Early echocardiographic screening within the first week of life enables prompt detection and follow-up of clinically relevant lesions. Given the lack of long-term follow-up in this study, ongoing surveillance is recommended to determine the natural history and prognostic significance of these findings. Integration of IDM cardiac screening into regional neonatal care policies should be considered in high-prevalence settings such as Saudi Arabia.

## Introduction

Diabetes mellitus complicates approximately 1-2% of pregnancies worldwide and is a major risk factor for adverse neonatal outcomes, particularly congenital heart diseases (CHDs). Infants of diabetic mothers (IDMs) are estimated to have a three- to five-fold higher risk of CHDs compared with those born to non-diabetic mothers [1]. A recent meta-analysis involving more than 46 million mother-child pairs confirmed that even gestational diabetes mellitus (GDM) increases the odds of CHD by 32% (95% CI: 1.21-1.45), with strong associations to atrial and ventricular septal defects (VSDs) [2].

In Saudi Arabia, diabetes in pregnancy represents a significant health challenge. A 2023 study in the Aseer region reported that 32.8% of pregnant women were affected by diabetes of various types (gestational, type 1, and type 2), and their infants demonstrated a markedly higher prevalence of cardiac anomalies [3]. Other regional studies have reported similar patterns, reflecting the substantial burden of maternal diabetes across the Gulf states.

The underlying pathogenesis of CHD in IDMs is multifactorial, involving genetic susceptibility, hyperglycemia-induced oxidative stress, and disruptions in cellular signaling during organogenesis. These insults predispose the fetus to both structural malformations (e.g., VSD, transposition of the great arteries, aortic stenosis, conotruncal defects) and functional changes such as hypertrophic cardiomyopathy [4,5].

King Fahad Hospital, Al Baha, was selected for this study as it is the sole tertiary referral hospital in the region, providing neonatal intensive care and echocardiographic services for high-risk pregnancies. While this single-center design limits generalizability, the hospital’s catchment area makes its data highly representative of the region’s population.

Echocardiographic screening within the first week of life is particularly valuable, as it allows for early detection of both structural CHDs and functional abnormalities such as septal hypertrophy. However, timing also introduces limitations, as some transitional findings (e.g., patent ductus arteriosus (PDA) and patent foramen ovale (PFO)) may resolve spontaneously in the early neonatal period. Distinguishing between physiological transitional changes and pathological anomalies is therefore crucial for correct interpretation [4,5].

To date, no study has examined the echocardiographic spectrum of IDMs in the Al Baha region. Furthermore, the impact of maternal diabetes type and glycemic control on neonatal cardiac outcomes remains incompletely described in local literature. This study aims to address this gap by evaluating the prevalence and spectrum of echocardiographic abnormalities in IDMs compared with controls at King Fahad Hospital.

## Materials and methods

This retrospective analytic study was conducted at the Neonatal Intensive Care Unit (NICU) and Newborn Nursery (NBN) of King Fahad Hospital in Al Baha City, Saudi Arabia, between January 2024 and June 2025. The study population comprised two groups: the case group included 100 neonates born to mothers diagnosed with either type 1 diabetes mellitus, type 2 diabetes mellitus, or gestational diabetes mellitus, while the control group consisted of 100 neonates born to non-diabetic mothers. Eligible participants were neonates whose mothers met the inclusion criteria and who underwent echocardiographic evaluation within the first seven days of life. Mothers with additional maternal risk factors, such as autoimmune diseases or drug-induced risks for congenital malformations, were excluded from the study.

Maternal and neonatal records were reviewed to obtain detailed demographic and clinical data. The maternal data included diabetes type, method of glycemic control, and any associated comorbidities. Neonatal data encompassed sex, birth weight, gestational age, Apgar scores at one and five minutes, mode of delivery, and whether NICU admission was required. Additional recorded clinical parameters included the presence of obstetric trauma, respiratory distress, tachycardia, cardiac murmurs, cyanosis, abdominal or neurological abnormalities, neonatal jaundice, and blood glucose levels within the first 24 hours of life.

All neonates underwent a comprehensive echocardiographic examination performed by a pediatric cardiologist within the first week after delivery. The assessment utilized M-mode, two-dimensional (2D), and Doppler imaging techniques to evaluate structural and functional cardiac status. The echocardiographic evaluation aimed to detect congenital heart diseases, assess chamber enlargement, identify pulmonary artery dilatation, evaluate ventricular function, and determine the presence of pulmonary hypertension, patent foramen ovale (PFO), and/or patent ductus arteriosus (PDA). Hypertrophic cardiomyopathy (HCM) was diagnosed when the ratio of the interventricular septum thickness to the posterior left ventricular wall exceeded 1:3. Atrial septal defects (ASDs) were classified as tiny and insignificant if their size was ≤ 3 mm and as small if ≤ 5 mm, which may be associated with a small left-to-right shunt [2].

Echocardiographic findings were categorized into three groups: structurally normal hearts, age-appropriate physiological findings such as PDA or PFO, and abnormalities requiring follow-up or intervention, including VSD and HCM [2].

Data analysis was performed using the SPSS Statistics version 21 (IBM Corp., Armonk, USA). Descriptive statistics were presented as frequencies and percentages for qualitative variables, and as mean ± standard deviation (SD) for quantitative variables. The Chi-square (χ²) test was used to compare categorical variables, while the Student’s t-test was applied to compare means between two independent groups. A p-value of less than 0.05 was considered statistically significant.

The study was approved by the Scientific Research Committee of King Fahad Hospital, Al-Baha (approval no. IRB-KFH14052025/2o).

## Results

Maternal characteristics

**Figure 1:**
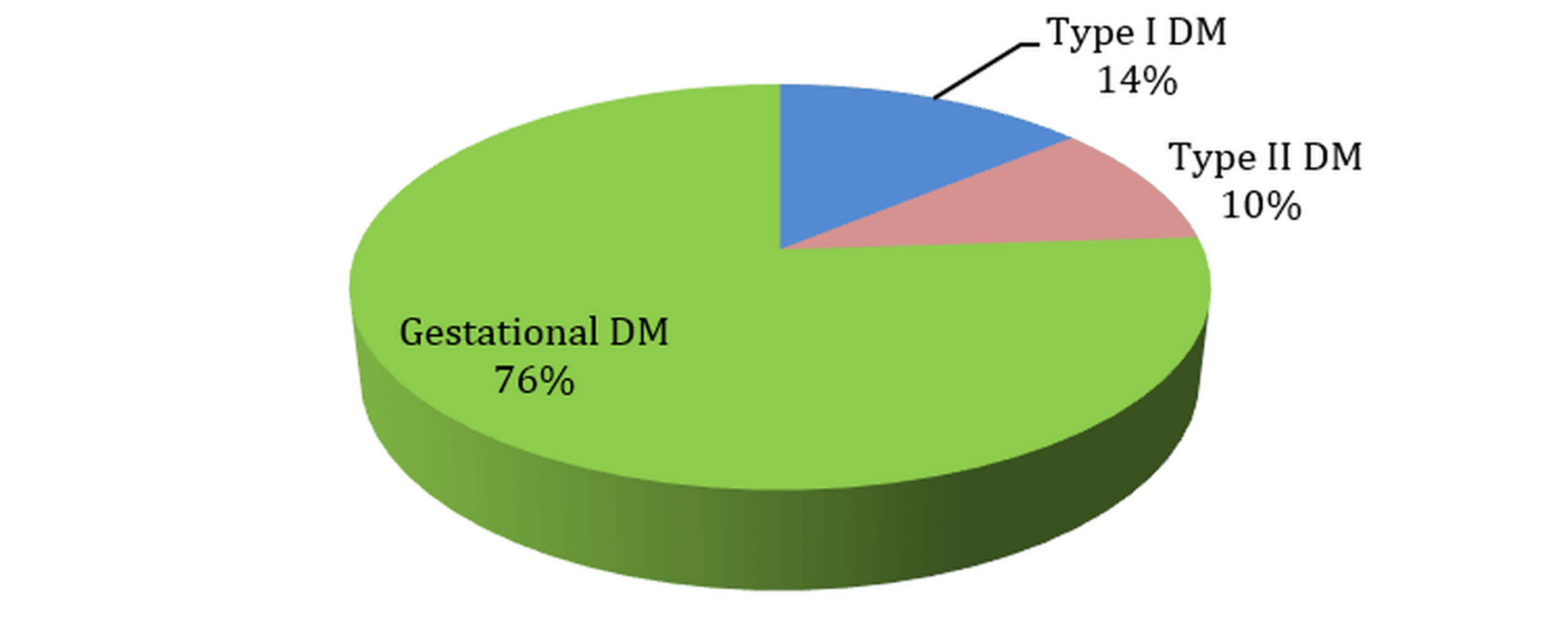
Type of Diabetes Mellitus (DM) Among the Studied Sample

The study included 100 diabetic mothers (IDMs). The mean maternal age was 34.3 ± 5.25 years (range: 23-46 years) (Table 1). Gestational diabetes was the most common type (76%), followed by type 1 DM (14%) and type 2 DM (10%) (Figure 1). Nearly half of the mothers (49%) had comorbid conditions, most frequently infections (29%), hypothyroidism (7%), and preeclampsia (5%).

**Table 1:** Characteristics of the Studied Mothers *Percentages may exceed 49% as some mothers had more than one comorbid condition. UTI: urinary tract infection; DM: diabetes mellitus

Characteristics	Number (N=100)	Percentage (%)
Age of Mothers (years)		
Mean ± SD	34.28 ± 5.25	
Range	23–46	
Type of Diabetes Mellitus		
Type 1 DM	14	14.0%
Type 2 DM	10	10.0%
Gestational DM	76	76.0%
Diabetes Management		
Diet Control	58	58.0%
Oral Hypoglycemics	12	12.0%
Insulin	30	30.0%
Presence of Comorbidities		
Yes	49	49.0%
No	51	51.0%
Type of Comorbidities*		
Infection (including UTI)	29	29.0%
Hypothyroidism	7	7.0%
Preeclampsia	5	5.0%
Polyhydramnios	4	4.0%
Obesity	4	4.0%
Bronchial Asthma	3	3.0%
Oligohydramnios	2	2.0%
History of Myomectomy	2	2.0%
Hypertension	2	2.0%
Diabetic Ketoacidosis	2	2.0%
Twin Pregnancy	2	2.0%
Cardiac Disease	2	2.0%

Neonatal characteristics

The mean gestational age of infants was 37.4 ± 1.6 weeks, with a mean birth weight of 2.97 ± 0.48 kg (Table 2). An equal number of male and female infants were observed (50% each). NICU admission was required in 22% of cases (Table 3), with respiratory distress (12%) and hypoglycemia (7%) being the leading causes. The median NICU stay was 5.5 days (range: 2-19 days).

**Table 2:** Characteristics of the Studied Babies *Age of the baby when echocardiography was performed.

Characteristics	Number (N=100)	Percentage (%)
Age of Baby at Echocardiography (days)*		
Mean ± SD	2.27 ± 0.96	
Range	1–7	
Sex of Baby		
Male	50	50.0%
Female	50	50.0%
Gestational Age (weeks)		
Mean ± SD	37.44 ± 1.63	
Range	29.0–40.0	
Weight of Baby (kg)		
Mean ± SD	2.97 ± 0.48	
Range	1.0–4.0	
Weight for Gestational Age (kg)		
Mean ± SD	2.85 ± 0.53	
Range	1.0–4.0	

**Table 3:** History of NICU Admission Among the Studied Babies NICU: neonatal ICU

Characteristics	Number (N=100)	Percentage (%)
NICU Admission		
Yes	22	22.0%
No	78	78.0%
Reasons for NICU Admission		
Respiratory Distress	12	12.0%
Hypoglycemia	7	7.0%
Hypoglycemia and Jaundice	1	1.0%
Poor Suckling	1	1.0%
Sepsis	1	1.0%
Duration of NICU Admission (days)		
Median	5.5	
Range	2.0–19.0	

Echocardiographic findings

**Figure 2:**
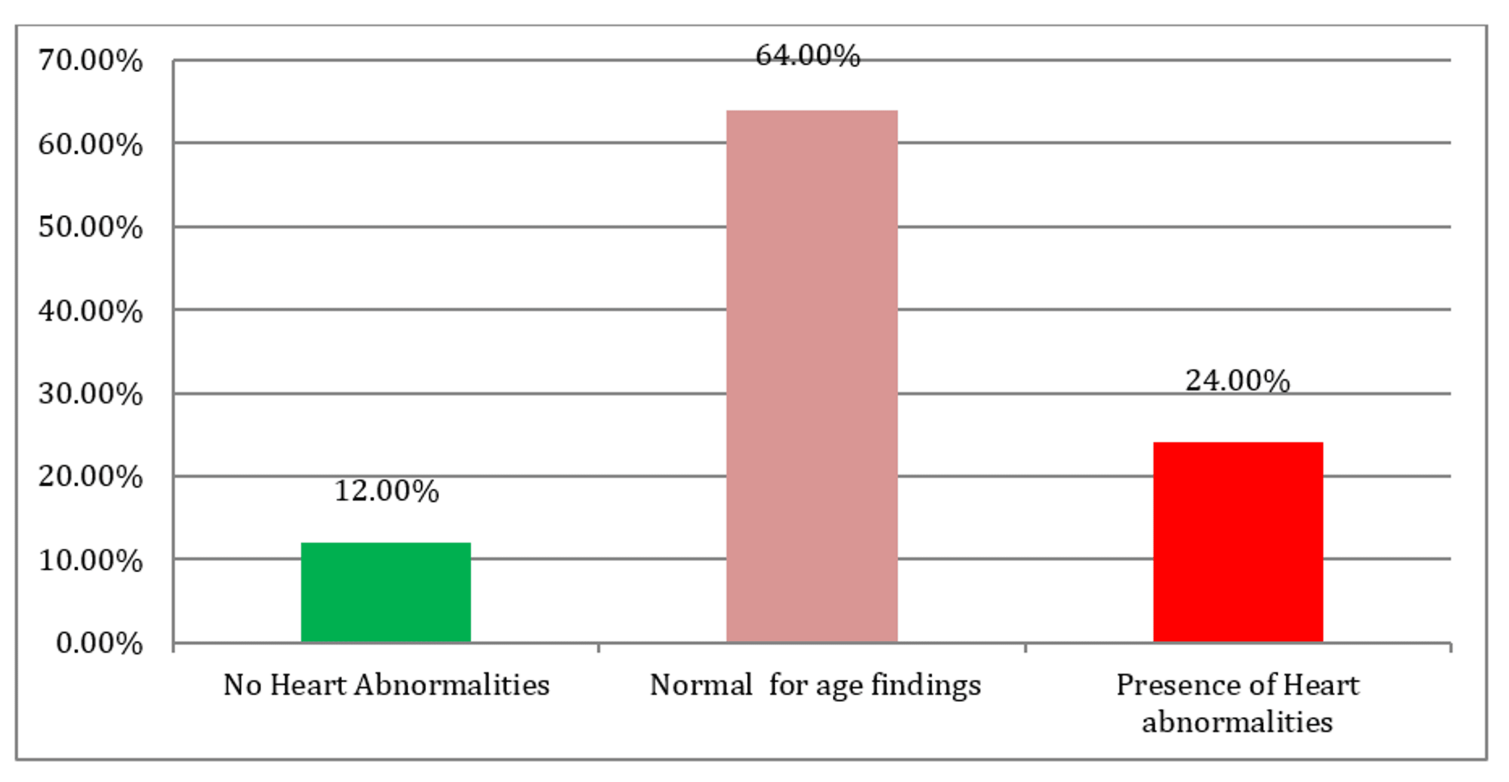
Congenital Heart Abnormalities Among the Studied Babies

**Figure 3:**
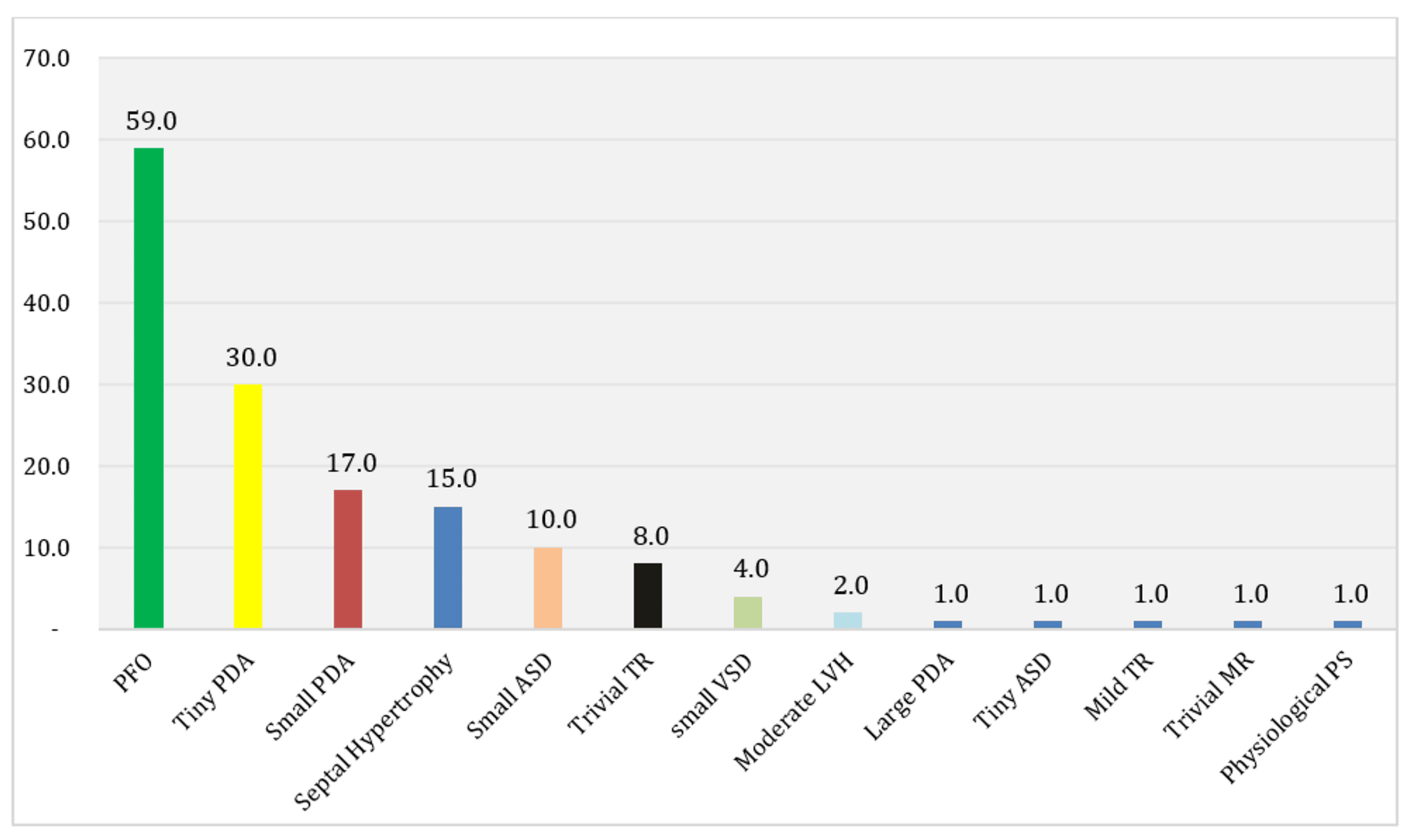
Percentages of Heart Findings Among the Studied Babies PFO: patent foramen ovale; PDA: patent ductus arteriosus; TR: tricuspid regurgitation; ASD: atrial septal defect; MR: mitral regurgitation; PS: pulmonary stenosis; VSD: ventricular septal defect; LVH: left ventricular hypertrophy

Echocardiography performed within the first week of life (mean 2.27 ± 0.96 days) revealed that 24% of infants had congenital heart abnormalities, while 64% had findings considered normal for age (such as PFO or small PDA), and only 12% showed completely normal cardiac anatomy. The most frequent pathological findings included: VSD, PDA, and HCM. Among the age-appropriate findings, isolated PFO and PFO with tiny PDA were the most prevalent (Table 4, Figure 2, Figure 3).

**Table 4:** Congenital Heart Abnormalities Among the Studied Babies PFO: patent foramen ovale; PDA: patent ductus arteriosus; TR: tricuspid regurgitation; ASD: atrial septal defect; MR: mitral regurgitation; PS: pulmonary stenosis; VSD: ventricular septal defect; LVH: left ventricular hypertrophy

Characteristics	Number (N=100)	Percentage (%)
Cardiac Findings		
No heart abnormalities	12	12.0%
Normal for age findings	64	64.0%
Presence of heart abnormalities	24	24.0%
Findings normal for age		
PFO alone	20	20.0%
Small PDA alone	7	7.0%
Tiny PDA alone	3	3.0%
Tiny PDA, PFO	18	18.0%
Small PDA, PFO	5	5.0%
Trivial TR, small ASD	3	3.0%
Trivial TR, PFO	3	3.0%
Trivial TR, small ASD, PFO	2	2.0%
Mild TR, trivial MR	1	1.0%
Tiny PDA, small ASD	1	1.0%
Physiologic PS, PFO, tiny ASD	1	1.0%
Presence of Heart abnormalities details		
Small VSD alone	2	2.0%
Tiny PDA, small VSD, PFO	2	2.0%
Large PDA, small ASD	1	1.0%
Tiny PDA, moderate LVH	1	1.0%
Small PDA, small ASD, PFO	2	2.0%
Septal hypertrophy	4	4.0%
Septal hypertrophy, PFO	4	4.0%
Septal hypertrophy, tiny PDA, PFO	2	2.0%
Septal hypertrophy, small PDA	2	2.0%
Septal hypertrophy, tiny PDA, PFO	2	2.0%
Septal hypertrophy, tiny PDA, small ASD	1	1.0%
Septal hypertrophy, LVH, small PDA	1	1.0%
Cardiac Management		
Nothing	12	12.0%
Follow up	87	87.0%
Medication	1	1.0%

For clarity, congenital heart abnormalities in this study refer to pathological lesions requiring follow-up or potential intervention. Transitional neonatal findings such as PFO, small PDA, and trivial regurgitant lesions were analyzed separately as normal-for-age physiological findings.

Association with maternal and neonatal factors

Analysis of maternal and neonatal variables in relation to the presence of congenital heart abnormalities demonstrated that affected infants underwent echocardiographic assessment significantly earlier than those without abnormalities (mean 1.91 ± 0.71 days versus 2.38 ± 1.00 days; p = 0.036). This earlier timing reflected clinician-triggered evaluation based on bedside concerns rather than protocol differences. Neonates with cardiac defects had a significantly lower weight-for-gestational-age compared to their unaffected counterparts (mean 2.62 ± 0.49 kg versus 2.90 ± 0.54 kg; p = 0.026). No statistically significant associations were observed between the occurrence of cardiac anomalies and maternal age, type of diabetes, or the need for NICU admission (p > 0.05) (Table 5).

**Table 5:** Distribution of Congenital Heart Abnormalities among the Studied Babies *Significant difference (p < 0.05). Categorical data is given as n (%). NICU: neonatal ICU; DM: diabetes mellitus

Characteristics	Presence of Congenital Heart Abnormalities (n=24)	No Congenital Heart Abnormalities (n=76)	p-value
Age of the mother (years): mean ± SD	34.67 ± 6.08	34.16 ± 5.01	0.682
Type of Diabetes Mellitus:			0.163
Type I DM	1 (4.1%)	13 (17.1%)	
Type II DM	4 (16.7%)	6 (7.9%)	
Gestational DM	19 (79.2%)	57 (75.0%)	
Diabetes Management:			0.250
Diet Control	17 (70.8%)	41 (53.9%)	
Oral hypoglycemic	1 (4.2%)	11 (14.5%)	
Insulin	6 (25.0%)	24 (31.6%)	
Presence of Co‑morbidity among mothers:			0.722
No	11 (45.8%)	38 (50.0%)	
Yes	13 (54.2%)	38 (50.0%)	
Sex of Baby:			0.349
Boy	14 (58.3%)	36 (47.4%)	
Girl	10 (41.7%)	40 (52.6%)	
Gestational Age (weeks): mean ± SD	37.58 ± 1.66	37.39 ± 1.63	0.625
Age of the baby during Echo investigation (days): mean ± SD	1.91 ± 0.71	2.38 ± 1.00	0.036*
Weight of the baby (kg): mean ± SD	2.96 ± 0.49	2.97 ± 0.48	0.976
Weight for Gestational Age (kg): mean ± SD	2.62 ± 0.49	2.90 ± 0.54	0.026*
NICU Admission:			0.874
Yes	5 (20.8%)	17 (22.4%)	
No	19 (79.2%)	59 (77.6%)	

Maternal diabetes management (diet, oral agents, insulin) and the presence of comorbidities were analyzed, but no significant correlations with specific cardiac anomalies were found. Given the modest sample size, further lesion-specific stratification was not pursued to avoid unstable estimates.

Correlations

Pearson correlation analysis (Table 6) revealed a significant negative correlation between gestational age and age at echocardiography (r = -0.35; p < 0.01), as well as between gestational age and duration of NICU stay (r = -0.56; p = 0.007).

**Table 6:** Correlation Between Different Study Variables ** Correlation is significant at the 0.01 level (2-tailed). NICU: neonatal ICU; echo: echocardiography

		Mother's age	Age of the baby when echo done	Gestational age	Weight of the baby	Weight for gestational age	Duration of NICU admission in days
Mother’s age	Pearson Correlation	1	.187	-.077	-.181	-.044	.075
	Sig. (2-tailed)		.063	.448	.071	.663	.740
	N	100	100	100	100	100	22
Age of the baby when echo done	Pearson Correlation	.187	1	-.352^**^	-.150	.045	.259
	Sig. (2-tailed)	.063		.000	.135	.659	.244
	N	100	100	100	100	100	22
Gestational age	Pearson Correlation	-.077	-.352^**^	1	.471^**^	.012	-.556^**^
	Sig. (2-tailed)	.448	.000		.000	.907	.007
	N	100	100	100	100	100	22
Weight of the baby	Pearson Correlation	-.181	-.150	.471^**^	1	-.153	-.341
	Sig. (2-tailed)	.071	.135	.000		.130	.121
	N	100	100	100	100	100	22
Weight for gestational age	Pearson Correlation	-.044	.045	.012	-.153	1	-.135
	Sig. (2-tailed)	.663	.659	.907	.130		.548
	N	100	100	100	100	100	22
Duration of NICU admission in days	Pearson Correlation	.075	.259	-.556^**^	-.341	-.135	1
	Sig. (2-tailed)	.740	.244	.007	.121	.548	
	N	22	22	22	22	22	22

## Discussion

The findings of this study are consistent with recent regional data on the prevalence and spectrum of congenital heart disease (CHD) in infants of diabetic mothers (IDMs). In a 2023 study from the Aseer region, PDA was reported in nearly 38% of IDMs, hypertrophic cardiomyopathy (HCM) in 37%, and VSD in 33%, closely mirroring the distribution observed in our study population. Similarly, a study from tertiary centers in Riyadh reported an overall CHD incidence of approximately 11% in IDMs, with PDA and PFO being the most frequently identified lesions [3,6].

Mechanistic evidence from a recent 2025 review underscores the role of maternal hyperglycemia in inducing oxidative stress, epigenetic dysregulation, and altered signaling pathways during cardiac organogenesis, particularly when diabetes is present before conception. These early gestational insults predispose the fetus to structural defects such as septal lesions and conotruncal anomalies [7,8].

By contrast, the Al-Ahsa cohort reported a much higher prevalence (68.3%) [9]. These differences may be explained by population heterogeneity, referral bias to tertiary centers, variations in maternal diabetes prevalence, and differences in diagnostic thresholds or echocardiographic protocols across regions.

For clarity, in this study, the term congenital heart abnormalities refers exclusively to structural pathological lesions requiring follow-up or potential intervention (e.g., VSD, ASD, significant PDA, HCM), while functional or transitional findings such as PFO, small PDA, and trivial regurgitant lesions were categorized separately as age-appropriate physiological changes. This distinction was made to avoid conflating transient physiological adaptations with true congenital defects.

In addition to structural abnormalities, functional changes have been documented in IDMs during the first postnatal week, including septal hypertrophy, prolonged myocardial performance index (MPI), reduced diastolic E/A ratio, and elevated pulmonary artery pressures [4]. Tissue Doppler echocardiography studies further reveal that IDMs, especially those born to mothers with poor glycemic control, exhibit early diastolic dysfunction [5,8].

Our findings of HCM and septal thickening are in line with these functional disturbances. The observed significant association between lower weight-for-gestational-age and CHD may reflect intrauterine maladaptation and hyperinsulinemia, both of which are well-documented contributors to abnormal cardiac development [4,5]. However, as our analyses were univariate, these associations should be interpreted cautiously, as residual confounding cannot be excluded. Larger studies with multivariable adjustment are needed to confirm these findings.

The correlation analysis was limited by the small number of neonates who required NICU admission (n = 22), which may reduce power and increase susceptibility to bias. Pearson correlation was applied because the continuous variables (gestational age, birth weight) showed approximate normal distribution; confidence intervals and Spearman sensitivity analyses were not pursued due to sample size constraints. The observed correlations have clear clinical relevance, as lower gestational age was associated with both longer NICU stay and earlier clinician-triggered echocardiography.

Effect-size estimates (odds ratios or relative risks with confidence intervals) and multivariable regression modeling were not undertaken due to event counts and power limitations; larger prospective studies are needed for stable effect estimates. Analyses therefore relied on predefined univariate comparisons without multiple-comparison adjustment, as only a limited set of variables was assessed.

The earlier echocardiography observed among affected infants likely reflects clinician-triggered imaging based on neonatal symptoms (e.g., murmur, respiratory distress) or bedside findings, rather than protocolized timing. In most cases, this earlier detection influenced the timing of diagnosis and initiation of follow-up, rather than acute management decisions.

The prevalence of CHD in our study population (24%) is consistent with some regional and international reports, although lower than the Al-Ahsa and Pakistani studies (68.3% and 52.5%, respectively) [9,10]. These differences highlight the heterogeneity of IDMs across settings, and may reflect both true variation and methodological differences. Our findings reinforce the teratogenic impact of maternal hyperglycemia. The association between low weight-for-gestational-age and CHD in our study mirrors previous reports [4,11], further emphasizing the need for targeted early screening in at-risk newborns. Notably, more than 86% of CHDs in IDMs were detected within the first week of life in a recent Saudi cohort [9], underscoring the importance of early postnatal echocardiography.

Interestingly, despite gestational diabetes being the predominant form in our study population, structural cardiac anomalies were still frequent. This aligns with findings from both the Al-Ahsa study [9] and the National Guard Health Affairs (NGHA) cohort in Riyadh [6], indicating that even when gestational diabetes is diagnosed later in pregnancy and managed appropriately, it may still significantly influence fetal cardiac development. This observation underscores the importance of optimizing maternal glycemic control as early as possible in pregnancy, ideally before conception.

The present study has several limitations. Its retrospective, single-center design limits the ability to establish causal relationships and may restrict generalizability. The sample size, although adequate for descriptive purposes, may be insufficient to detect subtle subgroup differences. Furthermore, the absence of long-term follow-up prevents assessment of the natural history and clinical outcomes of the identified cardiac abnormalities. Nevertheless, these limitations are consistent with other local studies in the field [6,9], and our study adds novel data from the Al Baha region, being the first to assess cardiac outcomes in IDMs with detailed echocardiographic evaluation.

Recommendations

Routine echocardiographic screening should be performed for all neonates of diabetic mothers within the first week of life. Prenatal diabetes care programs must prioritize optimal glycemic control before conception and during early pregnancy. Screening recommendations should also be aligned with international consensus statements, such as those from the American Heart Association and European Society of Cardiology, which advocate close monitoring of IDMs. Larger, multicenter prospective studies with follow-up are needed to clarify the long-term outcomes of cardiac findings in this high-risk group. Equally important, structured long-term surveillance programs should be implemented to monitor persistence, resolution, or progression of cardiac abnormalities detected in the neonatal period, ensuring early intervention when clinically indicated.

## Conclusions

In this study, 24% of infants of diabetic mothers were found to have congenital heart disease, with structural anomalies such as ventricular septal defect, patent ductus arteriosus, and hypertrophic cardiomyopathy being the most clinically significant findings that justify routine echocardiographic screening. In contrast, transitional neonatal changes such as PFO, small PDA, and trivial regurgitant lesions were considered physiological and not overinterpreted as pathological CHDs.

While gestational diabetes was the predominant maternal diagnosis, structural anomalies were observed across all diabetes types, highlighting that the risk is not confined to a single subgroup. Early echocardiography within the first week of life enabled timely detection and initiation of follow-up, though most cases required observation rather than acute intervention.

The retrospective single-center design, modest sample size, and lack of long-term follow-up limit the generalizability of these results. Nevertheless, our findings support the integration of echocardiographic screening for infants of diabetic mothers into regional neonatal care protocols in Saudi Arabia, where maternal diabetes is highly prevalent.

Future research should focus on longitudinal follow-up to establish the prognostic significance of both structural and functional findings, as well as mechanistic studies and maternal metabolic profiling to better define risk pathways.
